# Essential oil-containing solutions (mouthwashes) preserve dental enamel with releasing low Ca and P concentrations without morphology alterations: an *in vitro* study

**DOI:** 10.3389/fchem.2024.1341769

**Published:** 2024-02-28

**Authors:** Sheila Cristina Almeida Neves Mutran, Paulo Roberto de Carvalho-Filho, Mara Eliane Soares Ribeiro, Kelson do Carmo Freitas Faial, Rafael Rodrigues Lima, Roberta Souza D’Almeida Couto

**Affiliations:** ^1^ Department of Dentistry, School of Dentistry, Federal University of Pará, Belém, Brazil; ^2^ Department of Dentistry, School of Dentistry, University of São Paulo, São Paulo, Brazil; ^3^ Environment Section, Evandro Chagas Institute, Ananindeua, Brazil; ^4^ Laboratory of Functional and Structural Biology, Institute of Biological Sciences, Federal University of Pará, Belém, Brazil

**Keywords:** oils, volatile, dental enamel, calcium, chromatography, scanning electron microscopy

## Abstract

**Introduction:** The use of natural products such as essential oils has been suggested due to their promising pharmacological effects and economic viability. This study aimed to determine hydrogenic potential (pH), titratable acidity (TA), and ion concentrations of five solutions containing essential oils (EO), when used as a EO-containing solutions, and evaluate ion concentrations, enamel surface loss, and morphology alterations in enamel.

**Materials and methods:** The pH, TA, calcium (Ca), potassium (K), and sodium (Na) concentrations of five EO-containing solutions were measured. Bovine enamel specimens were submitted to two daily 30-s immersions in artificial saliva, citric acid, distilled water, BaCloTea (Basil, Clove e Tea Tree), GeLaTeaPep (Geranium, Lavender, Tea Tree and Peppermint), EucaLem (Eucalyptus and Lemon), Cinnamon, or Spearmint solutions for 14 days. Ca, K, Na, and phosphorus (P) were quantified through ions chromatography, enamel surface loss was determined by profilometry, and surface morphology was qualitatively analyzed through scanning electron microscopy. Data were submitted to one-way ANOVA and Tukey (*p* < 0.05).

**Results:** The five EO-containing solutions presented significantly lower pH values than distilled water (*p* < 0.05). The GeLaTeaPep group presented a significantly higher TA value than BaCloTea (*p* < 0.05), which in turn showed a significantly higher TA value than the other solutions (*p* < 0.05). The distilled water presented significantly higher Ca, K, and Na concentrations than all EO-containing solutions (*p* < 0.05). The enamel exposed to EO-containing solutions showed lower Ca and P concentrations than artificial saliva (control) as well as significantly higher surface loss; however, the surface morphology was similar to the artificial saliva.

**Conclusion:** EO-containing solutions have low pH, TA, and low concentrations of Ca, Na, and K. Moreover, enamel exposed to these solutions showed low Ca and P concentrations and slight surface loss without morphology alteration.

## 1 Introduction

Aromatic plants have been used in cosmetics and folk medicine for many years; however, plant extracts and essential oils (EO) became attractive for the industry in the last decade since consumers seek sustainable therapeutics agents with possible reduced adverse effects (Winska et al., 2019; Ramsey et al., 2020).

The EOs are composed of volatile metabolites secondary extracted from different parts of plants (flowers, buds, seeds, leaves, twigs, bark, herbs, wood, fruits, and roots) and present anticancer, antimicrobial, antiviral, antioxidant, and antibiotic properties (Aziz et al., 2018; Basavegowda et al., 2020; Ramsey et al., 2020). In dentistry, some EO-containing solutions have shown anxiolytic, antimicrobial, antiplaque, antigingivitis, and anticaries effects against oral pathogens (Zero et al., 2004; Dagli et al., 2015; Yadav et al., 2016; Quintas et al., 2017; Ghaderi and Solhjou, 2020).

Current lifestyle and diet habits may lead to tooth wear in young populations (Lussi et al., 2004; Donovan et al., 2021) and the effects of daily-use oral hygiene products on dental tissues need to be addressed. EO-containing mouthrinses do not require professional prescription or application, and are freely sold in supermarkets, drugstores, convenience stores, and on the internet; therefore, these over-the-counter products represent one of the most rapidly growing sectors of the oral care industry (Jardim et al., 2009; Weijden et al., 2015; Favaro et al., 2020). It is expected that products based on natural compounds such EOs may present limited detrimental effects. Some clinical trials have shown a significant reduction in plaque and gingivitis promoted by daily use of an EO-containing mouthrinse in combination with regular brushing and flossing (Sharma et al., 2004; Araujo et al., 2015; Alshehri, 2018). For a better understanding of the present study, it is worth highlighting that the use of the term essential oil-containing solutions mentioned throughout the text refers to mouthwashes.

This study aimed to determine the pH, TA, and mineral concentrations of EO-containing solutions, as well as their effect on enamel surface loss, morphology, and mineral concentrations.

## 2 Materials and methods

In the present *in vitro* study, available essential oil mouthwashes commercially (dōTERRA Cosméticos do Brasil Ltda.), BaCloTea (Basil, Clove e Tea Tree), GeLaTeaPep (Geranium, Lavender, Tea Tree and Peppermint), EucaLem (Eucalyptus and Lemon), Cinnamon, and Spearmint solutions were tested according to the chemical characterization of the mouthwashes in relation to pH, TA; and concentrations of Ca, K and Na by induced plasma optical emission spectrometry (ICP OES). In addition, tests of these rinses were carried out on the surface of bovine tooth enamel by: quantifying C, K, Na and P through ion chromatography; analysis of morphology by scanning electron microscopy (SEM); and loss of enamel surface dental by profilometry. Artificial saliva was used as a positive control group, acid citrus 0.3% as a negative control group. Distilled water and other mouthwashes mouthpieces of OEs were the experimental groups. The sample size was determined according to a previously carried out pilot study. As it is an *in vitro* study, this research did not follow a guideline.

### 2.1 Preparation of EO-containing solutions (mouthwashes)

Five essential oil mouthwashes (EOs) were handled individually or associated according to the manufacturer’s recommendations. The description of the groups, plants and main components of each EO, according to information from gas chromatography and mass spectrometry (GC/MS) made available by the company, are described in Table 1. The EO mouthwashes were prepared with the same distilled water of the control group.

**TABLE 1:** Description of the groups.

Group	Scientific name/main components^a^	Composition^b^
Artificial saliva	Artificial saliva	0.35% citric acid; 0.007% sodium chloride; 0.005% magnesium chloride; 0.1% potassium; 0.01% xylitol; 0.02% calcium carbonate; 1000 mL water	
Citric acid	2-hydroxypropane-1,2,3-tricarboxylic acid	0.3% citric acid	
Distilled water	Distilled water	Distilled water	
BaCloTea (Basil, Clove e Tea Tree)	*- Ocimum basilicum*/linalool, 1,8-cineol, eugenol, trans-α-bergamotene *- Eugenia caryophyllata*/eugenol, Eugenyl acetate, β-caryophyllene *- Melaleuca alternifolia*/terpinen-4-ol, ɣ-terpineno, α-terpineno	50 µL basil 50 µL cloven 50 µL tea tree 60 mL distilled water	
GeLaTeaPep (Geranium, Lavender, Tea Tree and Peppermint)	*- Pelargonium graveolens*/citronellol, citronellol format, geraniol, isomenthone *- Lavandula angustifolia*/linalyl acetate, linalool, lavandulyl acetate *- Melaleuca alternifolia*/terpinen-4-ol, ɣ-terpinene, α-terpinene *- Mentha piperita*/menthol, menthone, menthyl acetate	50 µL geranium 50 µL lavender 50 µL tea tree 50 µL peppermint 60 mL distilled water	
EucaLem (Eucalyptus and Lemon)	*- Eucalyptus radiata*/1,8-cineole, α-terpineol, limonene *- Citrus limon*/limonene, β-pinene, ɣ-terpinene	50 µL eucalyptus 50 µL lemon 60 mL distilled water	
Cinnamon (Cinnamon)	*Cinnamomum verum*/trans-cinnamaldehyde, trans-cinnamyl acetate, β-caryophyllene	50 µL cinnamon bark 60 mL distilled water	
Spearmint (Spearmint)	*Mentha spicata*/carvone, limonene, 1,8-cineole	50 µL spearmint 60 mL distilled water	

^a^ Gas chromatography-mass spectrometry data reported by the manufacturer.

^b^ The manufacturer declares that 1 drop corresponds to 50 µL.

### 2.2 Chemical characterization of EO-containing solutions

Five EO-containing solutions were manipulated according to the manufacturer’s recommendations; in addition, artificial saliva and 0.3% citric acid were respectively used as positive and negative controls (Table 1).

A total of 10 mL of each solution was added to a glass beaker and the pH value at 25°C was measured by a benchtop electrode previously calibrated with standard solutions (pH 4.01, 7.00, and 10.00). The electrode was washed with distilled water between each one of the three measurements.

Aliquots of 0.1 M sodium hydroxide were added to 10 mL of each solution (eight samples per group), and the total base volume (µL) of base needed to raise the initial pH to 7 was recorded. The TA was not determined for solutions with initial pH above 7 or neutral.

A total of 0.5 mL of each EO-containing solution was mixed with 0.5 mL of 10% (v/v) nitric acid (six samples per group), sieved with filter paper, diluted in 1:10 deionized water, and placed into polyethylene bottles (Medeiros et al., 2020). Ca^2+^, K^+^, and Na^+^ ions were quantified by using inductively coupled plasma optical emission spectrometry (ICP-OES) under axial configuration, equipped with an automatic sampling system (Vista-MPX CCD Simultaneous; Varian, Mulgrave, Australia), and operating conditions determined by specific software (ICP Expert Vista; Varian).

### 2.3 Effect of EO-containing solutions on enamel: ion concentrations, enamel surface loss, and morphology

Bovine incisors (*Bos taurus indicus*) were disinfected into 0.1% thymol solution for 1 week, scraped with periodontal curettes, brushed with pumice paste, and ultrasonically cleaned with distilled water for 10 min. The teeth with enamel cracks or defects were excluded after observation under a stereomicroscope with ×80 magnification, while sound teeth were stored for up to 30 days in distilled water at 4°C (ISO/TR 11405:1994 standards).

A total of 158 enamel specimens (4 × 4 × 2 mm) were obtained from the buccal surface of the teeth by using a water-cooled low-speed diamond saw (Isomet; Buehler, Lake Bluff, IL, United States of America) and ultrasonically cleaned with distilled water for 2 min (Machado et al., 2019). Seventy-two specimens were ground flat and wet-polished at 300 rpm for 1 min with aluminum oxide papers (400-, 600-, 1200-, and 4000-grit) and 1-μm-grit diamond paste) and ultrasonically cleaned with distilled water for 3 min in between every polishing step. Two areas of 72 specimens with curvature <0.3 µm were covered with an adhesive unplasticized polyvinyl chloride (UPVC) tape and an approximately 4 × 1 mm window was left exposed for further analysis of enamel surface loss.

Eight groups (BaCloTea, GeLaTeaPep, EucaLem, Cinnamon, Spearmint, Artificial saliva, Citric acid and Distilled water) were randomly assigned in accordance to different solutions in which the specimens were twice a day immersed under agitation for 30 s in solutions and then washed with distilled water. The specimens were stored in the artificial saliva in between exposure cycles, except for the distilled water group. The exposure cycles were repeated for 14 days to simulate the complete consumption of a mouthrinse bottle (Zero et al., 2004; Vieira-Junior et al., 2019).

The chemical quantification of the specimens was carried out after removing the dentin layer from 72 specimens that were divided into 9 experimental groups, in which 3 crushed specimens formed 1 sample (*n* = 3), using a tapered round-edge diamond bur mounted in a water-cooled high-speed air turbine, the enamel was ground, autoclaved, and placed in polytetrafluoroethylene vials previously decontaminated in nitric (1%, v/v) acid and washed with deionized water. Approximately 0.1 g of ground enamel, 3 mL of nitric acid, 1 mL of hydrochloric acid, and 1 mL of hydrogen peroxide were mixed and subjected to acidic digestion by microwave radiation (MARSXpress; CEM, Matthews, NC, United States). The samples were transferred to polypropylene bottles with a final volume of 50 mL and diluted (303x approximately) to quantify Ca, K, Na, and P through ions chromatography (ICS-2000 Dual; Dionex, Sunnyvale, CA, United States) (Petta et al., 2017).

**FIGURE 1:**
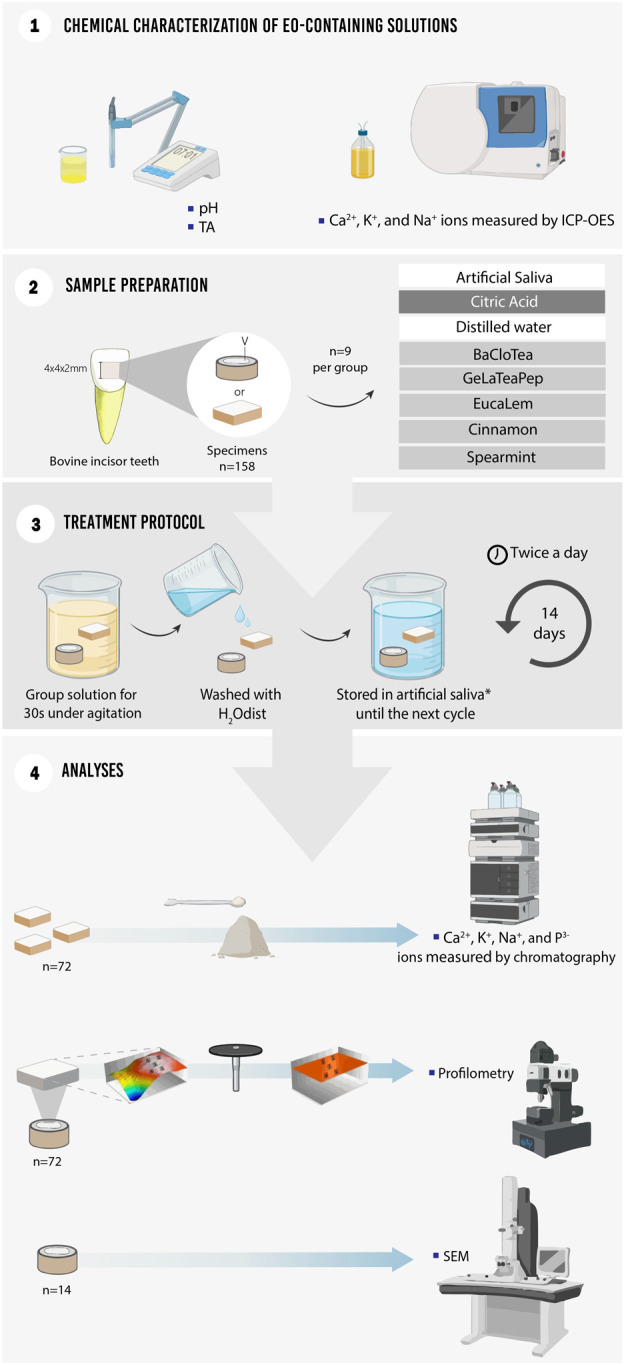
Schematic figure illustrating the study set-up.

The enamel surface loss was determined through profilometric measurements after 14-day exposure to the solutions (*n* = 9). In each specimen, the UPVC tapes were removed and these two areas were used as references. A 2-mm long (X) and 1-mm wide (Y) scanned that included both exposed and reference areas were performed by using an optical profilometer (Proscan 2000; Scantron, Taunton, United Kingdom) (Figure 1). The profilometer scanned two hundred 0.01-mm steps and ten 0.1-mm steps in the X- and *Y*-axis, respectively. The depth of the exposed area was determined by subtracting the average height of the two reference areas (3-point height tool) (Proscan Application Software 2.0.17) (Lopes et al., 2020).

Two random specimens per group (except for distilled water) were ultrasonically cleaned, dried, mounted on aluminum stubs, sputter-coated with gold-palladium (JFC 1100E Ion Sputter, Jeol), and the enamel surface morphology was qualitatively analyzed under a scanning electron microscope (SEM; VEGA3 Tescan, Brno, Czech Republic) at ×6,000 and ×10,000 magnifications.

### 2.4 Statistical analysis

The Shapiro-Wilk and Levene tests were used to respectively assess the normal distribution and homoscedasticity of pH, TA, ions concentration, and enamel surface loss data. Groups were compared using one-way analysis of variance (ANOVA) and *post hoc* Tukey multiple comparisons at a significance level of *p* < 0.05 (SPSS 17.0, IBM Corp., Chicago, IL, United States). The chromatography data were descriptively analyzed through means and standard deviations.

## 3 Results

### 3.1 Essential oil-containing solutions have low pH and titratable acidity

**FIGURE 2:**
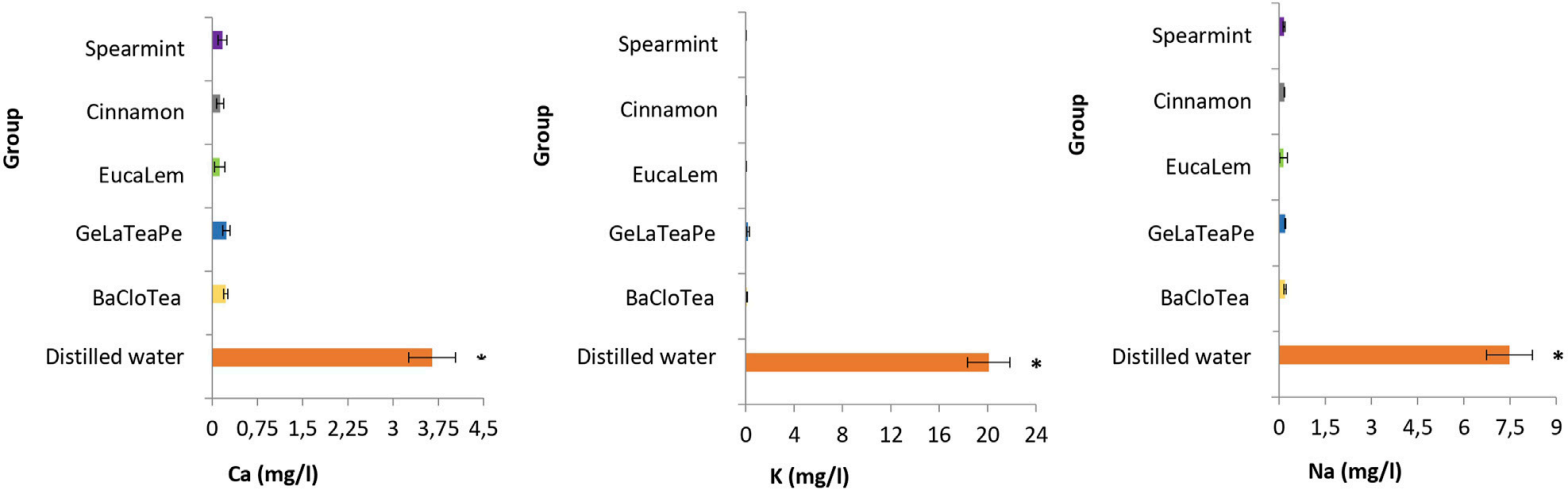
Ca, K, and Na concentrations (mg/L) in EO-containing solutions and distilled water. Groups followed by an asterisk (*) are significantly different from the other groups (ANOVA and Tukey, *p* < 0.05).

The five EO-containing solutions presented significantly lower pH than distilled water (*p* < 0.05) and below the critical value (5.5) for enamel dissolution. BaCloTea (Basil, Clove e Tea Tree), GeLaTeaPep (Geranium, Lavender, Tea Tree and Peppermint), EucaLem (Eucalyptus and Lemon), and Cinnamon groups showed the lowest pH values. The pH and TA mean values for each group are shown in Table 2. The GeLaTeaPep group presented a significantly higher TA value than BaCloTea (*p* < 0.05), which in turn showed a significantly higher TA value in comparison to the other EO-containing solutions (*p* < 0.05). The distilled water presented significantly higher Ca, K, and Na concentrations than all EO-containing solutions (*p* < 0.05) (Figure 2).

**TABLE 2:** pH and AT mean values (± standard deviations) for EO-containing solutions and distilled water.

Group	Baseline pH*	TA (µL)**
BaCloTea	3.3 (±0.45)^a^	11.0 (±0.00)^D^
Cinnamon	3.4 (±0.43)^a^	8.8 (±0.35) ^AC^
GeLaTeaPep	3.4 (±0.56)^a^	21.3 (±2.61)^E^
EucaLem	3.7 (±0.20) ^ab^	8.0 (±1.48)^A^
Spearmint	4.3 (±0.57)^b^	6.7 (±1.28) ^AB^
Distilled water	7.7 (±0.46)^c^	**-**

* Groups with the same superscripted lowercase letter are not significantly different (one-way ANOVA, and Tukey, *p* < 0.05).

** Groups with the same superscripted capital letter are not significantly different (one-way ANOVA, and Tukey, *p* < 0.05).

### 3.2 EO-containing solutions release small concentrations of Ca and P

The Ca, K, Na, and P concentrations in enamel after 14-day exposure to EO-containing solutions are described in Table 3. Artificial saliva (positive control) and citric acid (negative control) presented the highest and the lowest Ca, K, Na, and P concentrations, respectively. GeLaTeaPep and EucaLem were the EO-containing solutions with the lowest Ca, Na, and P concentrations. All EO-containing solutions showed lower Ca and P concentrations than artificial saliva. Except for GeLaTeaPep and EucaLem groups, the other EO-containing solutions showed higher Ca and P concentrations than citric acid.

**TABLE 3:** Ca, K, Na, and P mean concentrations (± standard deviations) in enamel after 14-day exposure to EO-containing solutions, citric acid, distiller water, and artificial saliva.

Group	Ca (mg/L)	K (mg/L)	Na (mg/L)	P (mg/L)
GeLaTeaPep	23098.8 (±3002.7)	189.8 (±20.8)	7119.4 (±815.3)	1432.0 (±157.5)
EucaLem	23623.0 (±1884.6)	184.3 (±12.8)	7407.8 (±1688.8)	1444.8 (±119.7)
Citric acid	23980.5 (±2632.1)	171.0 (±24.9)	8193.5 (±779.3)	1497.9 (±107.7)
Cinnamon	24282.4 (±5917.2)	201.0 (±10.2)	8223.8 (±2002.6)	1465.1 (±307.1)
BaCloTea	25070.1 (±3937.3)	212.3 (±10.7)	8108.5 (±2119.7)	1613.3 (±226.7)
Spearmint	25919.9 (±6083.8)	195.0 (±19.0)	9367.8 (±2427.1)	1558.7 (±345.4)
Distilled water	27894.5 (±4752.0)	180.3 (±11.0)	9241.7 (±2464.2)	1771.0 (±218.3)
Artificial saliva	29322.6 (±2250.8)	231.2 (±51.9)	10778.1 (±1742.7	1818.3 (±142.4)

### 3.3 EO-containing solutions induce enamel surface loss without morphology alteration

All EO-containing solutions induced significantly higher enamel surface loss than artificial saliva and were not different from distilled water (except for the BaCloTea group) (Table 4). BaCloTea and Cinnamon groups showed the highest enamel surface loss and were not different from citric acid. EucaLem, GeLaTeaPep, and Spearmint groups present the lowest enamel surface loss.

**TABLE 4:** Means (± standard deviations) of enamel surface loss after 14-day exposure to EO containing solutions, citric acid, distiller water, and artificial saliva.

Group	Enamel surface loss (µm)
Artificial saliva	0.3 (±0.3)^a^
Distilled water	0.7 (±0.5) ^abc^
EucaLem	1.0 (±0.3)^b^
GeLaTeaPep	3.1 (±1.9)^b^
Spearmint	3.9 (±2.3) ^bc^
Cinnamon	5.3 (±2.9) ^cd^
BaCloTea	7.4 (±3.5)^d^
Citric acid	8.3 (±2.7)^d^

*Groups with the same superscripted lowercase letter are not significantly different (one-way ANOVA, and Tukey, *p* < 0.05).

**FIGURE 3:**
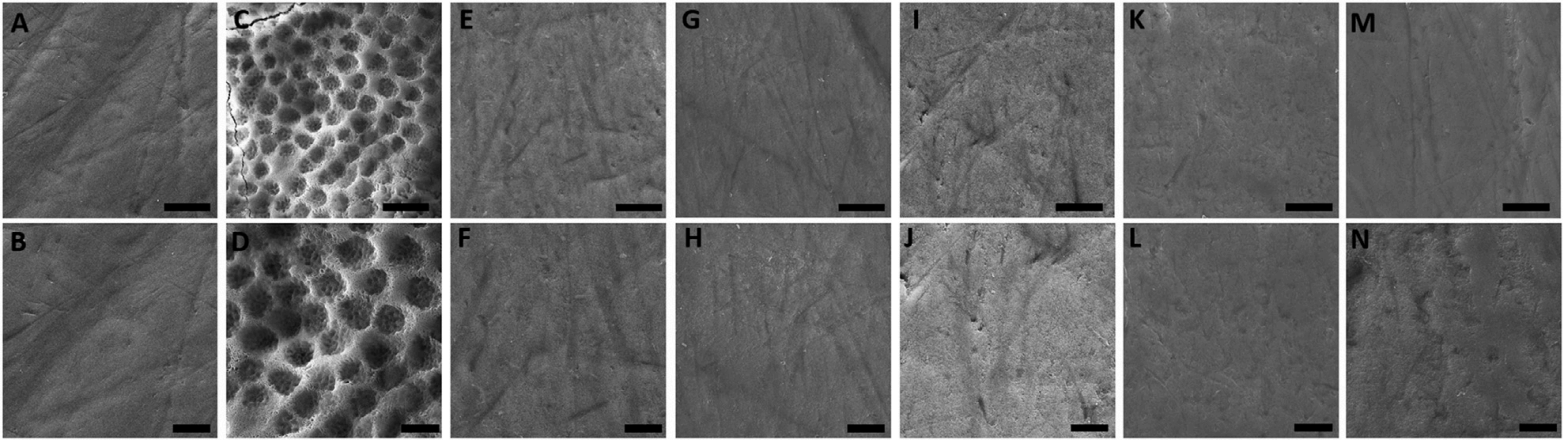
SEM photomicrographs (×6,000 and ×10,000 magnifications) of enamel after 14-day exposure to **(A, B)** artificial saliva, **(C, D)** citric acid, **(E, F)** BaCloTea, **(G, H)** GeLaTeaPep, **(I, J)** EucaLem, **(K, L)** Cinnamon, and **(M, N)** Spearmint.

The morphology of the enamel surface only exposed to artificial saliva was found predominantly smooth, regular, and uniform. Although slight marks of specimen preparation can be observed, the surface still presented aprismatic enamel without prisms exposure (Figures 3A, B). Conversely, the exposure to 0.3% citric acid resulted in the exposure of enamel prisms, interprismatic regions, and grooves that indicate accentuated demineralization (Figures 3C, D). The enamel surface of the specimens exposed to EO-containing solutions showed similar morphology of the positive control group (artificial saliva) (Figures 3E–N).

## 4 Discussion

The EO-containing solutions investigated in this study have low pH, TA, and Ca, K, and Na concentrations. In addition, these solutions released low Ca and P concentrations into the enamel and induced different levels of surface loss without affecting remarkable morphology alterations.

The EO-containing solutions presented pH values below 5.5, which is widely considered critical for enamel dissolution; however, this process also depends on the buffer capacity, TA, and Ca, P, and fluoride (F) concentrations (Dawes, 2003; Saads Carvalho and Lussi, 2020; Harper et al., 2021). Several oral care products such as F-containing mouthrinses have a low pH (Hellwig and Lussi, 2006). The results of this study are in agreement with other investigations on EO- and F-containing mouthrinses (Zero et al., 2004; Delgado et al., 2018; Valdivia-Tapia et al., 2021) and pH values similar to sports drinks (Sato et al., 2021). The EO-containing solutions investigated in this study showed low pH; however, they needed a small amount of base to be neutralized (low TA) (Saads Carvalho and Lussi, 2020), which represents a low erosive potential for tooth tissues (Harper et al., 2021). Delgado et al. (2018) observed some commercially available mouthrinses (Listerine Total Care, Listerine Ultraclean, Listerine Original, and Scope Classic) with pH below 5.5 and high TA; thus, both parameters were significantly correlated to dentin loss and erosive potential. Similar findings were also reported by Cavalcanti et al. (2010) regarding other branded mouthrinses.

In this study, the EO-containing solutions presented ions that are important for the oral environment. Ca is essential for remineralization, while P and Na play a major role in membrane potential and hypersensitivity regulation (Cummins, 2009; González-Cabezas and Fernández, 2018; Li et al., 2020). Moreover, Ca and P are the main components of hydroxyapatite [Ca_10_(PO_4_)_6_(OH)_2_]. The Spearmint showed the highest pH value, lowest TA, and Ca concentration similar to the other EO-containing solutions. This EO has 58% of carvone (C_10_H_14O_), which is a monoterpene ketone with promising antimicrobial, antispasmodic, anti-inflammatory, antioxidant, antinociceptive, and anticonvulsant activities (Dehsheikh et al., 2020; Pina et al., 2022); in addition, Mentha spicata has demonstrated to retard biofilm formation and can be considered an effective, cheap and safe alternative to improve oral health (Rasooli et al., 2009; Wiwattanarattanabut et al., 2017).

Among the scarce evidence on the effect of EO-containing mouthrinses, their use against cariogenic bacteria (Almeida Freires et al., 2015; Braga et al., 2019; Ma et al., 2020) and to reduce plaque and gingivitis have been suggested (Haas et al., 2016; Richards, 2017; Lynch et al., 2018). Some vegetable oils have been demonstrated to form a lipid-rich pellicle that minimizes enamel and dentin demineralization (Buchalla et al., 2003; Kensche et al., 2013; Ionta et al., 2017). The preventive potential of vegetable oils has been widely studied since they are natural, edible, cheap, and worldwide accessible (Ionta et al., 2017).

In this study, the 14-day protocol of exposure of enamel specimens to EO-containing solutions simulated two daily mouthrinses (morning and afternoon) usually performed by individuals (Vieira-Junior et al., 2019) and evaluated the sole effect of five EO-containing solutions on enamel without other variables such as erosion, wear, or toothbrushing (Zero et al., 2004; Delgado et al., 2018).

Most commercially available EO-containing mouthrinses usually contain 5%–27% of alcohol, which acts as a preservative, germicide, and solubilizer to maintain EO bioavailability (Silverman and Wilder, 2006; Gandini et al., 2012; Lynch et al., 2018; Pelino et al., 2018). The five EO-containing solutions evaluated in this study were prepared without the addition of alcohol by following the manufacturer’s recommendations for at-home use. Some studies have shown reliable antibacterial and antiplaque effects of alcohol-free EO-containing mouthrinses (Quintas et al., 2017; Lynch et al., 2018).

The highest Ca, K, Na, and P concentrations were observed in the enamel specimens exposed to artificial saliva (positive control). Despite the variety of artificial saliva formulations used for *in vitro* research, this product usually induces greater enamel remineralization than water (Ionta et al., 2014). The chromatography revealed that the EO-containing solutions released smaller amounts of Ca^2+^ and P ions when compared to the distilled water group.

Except for the negative control, the BaCloTea group showed the greatest enamel surface loss, which is in agreement with its lowest pH among the investigated EO-containing solutions. EucaLem, GeLaTeaPep, and Spearmint showed the lowest enamel surface loss mean values and were not significantly different from the distilled water group. However, in the SEM photomicrographs, the enamel surfaces of the specimens exposed to the EO-containing solutions were found similar to the positive control and distilled water groups. Loss of enamel surface was only clearly observed in the negative control group. While SEM is a method for qualitative analysis of the enamel surface microanatomy (Zhang et al., 2000), profilometry is a quantitative method that can accurately determine surface losses greater than 0.4 µm (Attin and Wegehaupt, 2014). Therefore, it can be assumed that profilometry can detect clinically imperceptible losses on the enamel surface that are not visualized through SEM.

Under slightly acidic pH, the Ca^2+^ ions from the tooth surface react with F ions to form a layer of calcium fluoride (CaF_2_) or CaF_2_-like molecule, which is more acid-resistant (Lussi et al., 2012; Schiffner, 2021). Interestingly, the EO-containing solutions investigated in this study showed chemical parameters that could benefit from the addition of F ions to remineralize the abovementioned enamel surface losses.

The effects of oral hygiene products on tooth structure must be well known, since they are widely used on a daily basis to maintain oral health. Thus, EO-containing solutions can be a viable and non-aggressive alternative to preserve tooth structure. Since this *in vitro* study did not fully simulate the oral environment, the effect of human saliva on acquired pellicle formation and enamel remineralization was not addressed (Nahsan et al., 2018; Lopes et al., 2020). Although the specimens were stored in artificial saliva, the effect of the EO-containing solutions on the enamel surface may have been more pronounced. Therefore, further *in situ* studies and clinical trials are encouraged to overcome these limitations.

The EO-containing solutions have low pH, TA, and low concentrations of Ca, Na, and K. Moreover, enamel exposed to these solutions showed low Ca and P concentrations and slight surface loss without morphology alteration.

## Data Availability

The original contributions presented in the study are included in the article/Supplementary material, further inquiries can be directed to the corresponding author.

## References

[B1] Almeida Freires N. Denny C. Benso B. Matias De Alencar S. Rosalen P. L. (2015). Antibacterial activity of essential oils and their isolated constituents against cariogenic bacteria: a systematic review. Molecules 20, 7329–7358. 10.3390/molecules20047329 25911964 PMC6272492

[B2] Alshehri F. A. (2018). The use of mouthwash containing essential oils (LISTERINE®) to improve oral health: a systematic review. Saudi Dent. J. 30, 2–6. 10.1016/j.sdentj.2017.12.004 30166864 PMC6112363

[B3] Araujo M. W. B. Charles C. A. Weinstein R. B. McGuire J. A. Parikh-Das A. M. Du Q. (2015). Meta-analysis of the effect of an essential oil–containing mouthrinse on gingivitis and plaque. J. Am. Dent. Assoc. 146, 610–622. 10.1016/J.ADAJ.2015.02.011 26227646

[B4] Attin T. S. Wegehaupt F. J. (2014). Methods for assessment of dental erosion. Monogr. Oral Sci. 25, 123–142. 10.1159/000360355 24993262

[B5] Aziz Z. A. A. Ahmad A. Setapar S. H. M. Karakucuk A. Azim M. M. Lokhat D. (2018). Essential oils: extraction techniques, pharmaceutical and therapeutic potential - a review. Curr. Drug Metab. 19, 1100–1110. 10.2174/1389200219666180723144850 30039757

[B6] Basavegowda N. Patra J. Baek K.-H. (2020). Essential oils and mono/bi/tri-metallic nanocomposites as alternative sources of antimicrobial agents to combat multidrug-resistant pathogenic microorganisms: an overview. Molecules 27, 1058. 10.3390/molecules25051058 PMC7179174 32120930

[B7] Braga A. S. Girotti L. D. Melo Simas L. L. Pires J. G. Pelá V. T. Buzalaf M. A. R. (2019). Effect of commercial herbal toothpastes and mouth rinses on the prevention of enamel demineralization using a microcosm biofilm model. Biofouling 35, 796–804. 10.1080/08927014.2019.1662897 31514534

[B8] Buchalla W. Attin T. Roth P. Hellwig E. (2003). Influence of olive oil emulsions on dentin demineralization *in vitro* . Caries Res. 37, 100–107. 10.1159/000069017 12652047

[B9] Cavalcanti A. L. Ramos I. A. Leite R. B. Oliveira M. C. Menezes K. M. Fernandes L. V. (2010). Endogenous pH, titratable acidity and total soluble solid content of mouthwashes available in the Brazilian market. Eur. J. Dent. 4, 156–159. 10.1055/s-0039-1697823 20396446 PMC2853830

[B10] Cummins D. (2009). Dentin hypersensitivity: from diagnosis to a breakthrough therapy for everyday sensitivity relief. J. Clin. Dent. 20, 1–9. 19489186

[B11] Dagli N. Dagli R. Mahmoud R. S. Baroudi K. (2015). Essential oils, their therapeutic properties, and implication in dentistry: a review. J. Int. Soc. Prev. Community Dent. 5, 335–340. 10.4103/2231-0762.165933 26539382 PMC4606594

[B12] Dawes C. (2003). What is the critical pH and why does a tooth dissolve in acid? J. Can. Dent. Assoc. 69, 722–724. 14653937

[B13] Dehsheikh A. B. Sourestani M. M. Dehsheikh P. B. Mottaghipisheh J. Vitalini S. Iriti M. (2020). Monoterpenes: essential oil components with valuable features. Mini Rev. Med. Chem. 20, 958–974. 10.2174/1389557520666200122144703 31969098

[B14] Delgado A. Ribeiro A. Quesada A. Rodríguez L. E. Hernández R. Wynkoop B. (2018). Potential erosive effect of mouthrinses on enamel and dentin. Gen. Dent. 66, 75–79. 29714705

[B15] Donovan T. Nguyen-Ngoc C. Abd Alraheam I. Irusa K. (2021). Contemporary diagnosis and management of dental erosion. J. Esthet. Restor. Dent. 33, 78–87. 10.1111/jerd.12706 33410255

[B16] Favaro J. C. Ribeiro E. Guiraldo R. D. Lopes M. B. Aranha A. M. F. Berger S. B. (2020). Effect of mouth rinses on tooth enamel surface. J. Oral Sci. 62, 103–106. 10.2334/josnusd.18-0370 31996511

[B17] Gandini S. Negri E. Boffetta P. La Vecchia C. Boyle P. (2012). Mouthwash and oral cancer risk quantitative meta-analysis of epidemiologic studies. Ann. Agric. Environ. Med. 19, 173–180. 22742785

[B18] Ghaderi F. Solhjou N. (2020). The effects of lavender aromatherapy on stress and pain perception in children during dental treatment: a randomized clinical trial. Complement. Ther. Clin. Pract. 40, 101182. 10.1016/j.ctcp.2020.101182 32891272

[B19] González-Cabezas C. Fernández C. E. (2018). Recent advances in remineralization therapies for caries lesions. Adv. Dent. Res. 29, 55–59. 10.1177/0022034517740124 29355426

[B20] Haas A. Wagner T. Muniz F. Fiorini T. F. Cavagni J. Celeste R. K. (2016). Essential oils-containing mouthwashes for gingivitis and plaque: meta-analyses and meta-regression. J. Dent. 55, 7–15. 10.1016/j.jdent.2016.09.001 27628316

[B21] Harper R. Shelton R. James J. Salvati E. Besnard C. Korsunsky A. M. (2021). Acid-induced demineralisation of human enamel as a function of time and pH observed using X-ray and polarised light imaging. Acta Biomater. 15, 240–248. 10.1016/j.actbio.2020.04.045 32438107

[B22] Hellwig E. Lussi A. (2006). Oral hygiene products and acidic medicines. Monogr. Oral Sci. 20, 112–118. 10.1159/000093358 16687890

[B23] Ionta F. Q. Alencar C. R. B. Val P. P. Boteon A. P. Jordão M. C. Honório H. M. (2017). Effect of vegetable oils applied over acquired enamel pellicle on initial erosion. J. Appl. Oral Sci. 25, 420–426. 10.1590/1678-7757-2016-0436 28877281 PMC5595115

[B24] Ionta F. Q. Mendonça F. L. De Oliveira G. C. De Alencar C. R. B. Honório H. M. Magalhães A. C. (2014). *In vitro* assessment of artificial saliva formulations on initial enamel erosion remineralization. J. Dent. 42, 175–179. 10.1016/J.JDENT.2013.11.009 24269764

[B25] Jardim J. J. Alves L. S. Maltz M. (2009). The history and global market of oral home-care products. Braz Oral Res. 23, 17–22. 10.1590/s1806-83242009000500004 19838554

[B26] Kensche A. Reich M. Kümmerer K. Hannig M. Hannig C. (2013). Lipids in preventive dentistry. Clin. Oral Investig. 17, 669–685. 10.1007/S00784-012-0835-9 23053698

[B27] Li H. Liu W. Zhou H. Sun Y. Zhang M. Wang J. (2020). *In vitro* dentine tubule occlusion by a novel toothpaste containing calcium silicate and sodium phosphate. J. Dent. 103, 100024. 10.1016/j.jjodo.2020.100024 34059302

[B28] Lopes R. Silva J. João-Souza S. H. Maximiano V. Machado A. C. Scaramucci T. (2020). Enamel surface loss after erosive and abrasive cycling with different periods of immersion in human saliva. Arc Oral Biol. 109, 104549. 10.1016/j.archoralbio.2019.104549 31541844

[B29] Lussi A. Hellwig E. Klimek J. (2012). Fluorides -mode of action and recommendations for use. Schweiz Monatsschr Zahnmed. 122, 1030–1042. 23192605

[B30] Lussi A. Jaeggi T. Zero D. (2004). The role of diet in the aetiology of dental erosion. Caries Res. 38, 34–44. 10.1159/000074360 14685022

[B31] Lynch M. C. Cortelli S. C. McGuire J. A. Zhang J. Ricci-Nittel D. Mordas C. J. (2018). The effects of essential oil mouthrinses with or without alcohol on plaque and gingivitis: a randomized controlled clinical study. BMC Oral Health 18, 6–10. 10.1186/s12903-017-0454-6 29321067 PMC5763666

[B32] Ma L. Chen J. Han H. Liu P. Wang H. Lin S. (2020). Effects of lemon essential oil and limonene on the progress of early caries: an *in vitro* study. Arc Oral Biol. 111, 104638. 10.1016/j.archoralbio.2019.104638 31901573

[B33] Machado A. Bezerra S. João-Souza S. H. Caetano T. M. Russo L. C. Carvalho T. S. (2019). Using fluoride mouthrinses before or after toothbrushing: effect on erosive tooth wear. Arc Oral Biol. 108, 104520. 10.1016/j.archoralbio.2019.104520 31445424

[B35] Medeiros T. L. M. Mutran S. C. A. N. Espinosa D. G. Do Carmo Freitas Faial K. Pinheiro H. H. C. D’Almeida Couto R. S. (2020). Prevalence and risk indicators of non-carious cervical lesions in male footballers. BMC Oral Health 20, 215. 10.1186/S12903-020-01200-9 32727438 PMC7392645

[B36] Nahsan F. Reis M. Francisconi-Dos-Rios L. F. Leão L. V. Paranhos L. R. (2018). Effectiveness of whitening mouthwashes on tooth color: an *in vitro* study. Gen. Dent. 66, e7–e10. 29513242

[B37] Pelino J. Passero A. Martin A. A. Charles C. A. (2018). *In vitro* effects of alcohol-containing mouthwashes on human enamel and restorative materials. Braz Oral Res. 32, e25. 10.1590/1807-3107bor-2018.vol32.0025 29561951

[B38] Petta T. M. Gomes Y. S. B. L. Esteves R. A. Faial K. C. F. Couto R. S. D. Silva C. M. (2017). Chemical composition and microhardness of human enamel treated with fluoridated whintening agents. A study *in situ* . Open Dent. J. 11, 34–40. 10.2174/1874210601711010034 28405245 PMC5368773

[B39] Pina L. Serafini M. Oliveira M. Sampaio L. A. Guimarães J. O. Guimarães A. G. (2022). Carvone and its pharmacological activities: a systematic review. Phytochemistry 196, 113080. 10.1016/j.phytochem.2021.113080 34999510

[B40] Quintas V. Prada-López I. Carreira M. J. Suárez-Quintanilla D. Balsa-Castro C. Tomás I. (2017). *In situ* antibacterial activity of essential oils with and without alcohol on oral biofilm: a randomized clinical trial. Front. Microbiol. 8, 2162. 10.3389/fmicb.2017.02162 29218030 PMC5703870

[B41] Ramsey J. T. Shropshire B. C. Nagy T. R. Chambers K. D. Li Y. Korach K. S. (2020). Essential oils and health. Yale J. Biol. Med. 93, 291–305. 32607090 PMC7309671

[B42] Rasooli I. Shayegh S. Astaneh S. D. A. (2009). The effect of mentha spicata and eucalyptus camaldulensis essential oils on dental biofilm. Int. J. Dent. Hyg. 7, 196–203. 10.1111/J.1601-5037.2009.00389.x 19659716

[B43] Richards D. (2017). Effect of essential oil mouthrinses on plaque and gingivitis. Evid. Based Dent. 18, 39–40. 10.1038/sj.ebd.6401233 28642553

[B44] Saads Carvalho T. Lussi A. (2020). Chapter 9: acidic beverages and foods associated with dental erosion and erosive tooth wear. Monogr. Oral Sci. 28, 91–98. 10.1159/000455376 31940633

[B45] Sato T. Fukuzawa Y. Kawakami S. Suzuki M. Tanaka Y. Terayama H. (2021). The onset of dental erosion caused by food and drinks and the preventive effect of alkaline ionized water. Nutrients 13, 3440. 10.3390/nu13103440 34684439 PMC8537624

[B46] Schiffner U. (2021). Use of fluorides for caries prevention. Bundesgesundheitsblatt - Gesundheitsforsch. Gesundheitsschutz. 64, 830–837. 10.1007/S00103-021-03347-4 PMC8241667 34115151

[B47] Sharma N. Charles C. Lynch M. Qaqish J. McGuire J. A. Galustians J. G. (2004). Adjunctive benefit of an essential oil–containing mouthrinse in reducing plaque and gingivitis in patients who brush and floss regularly: a six-month study. J. Am. Dent. Assoc. 135, 496–504. 10.14219/jada.archive.2004.0217 15127875

[B48] Silverman S. Wilder R. (2006). Antimicrobial mouthrinse as part of a comprehensive oral care regimen: safety and compliance factors. J. Am. Dent. Assoc. 137, 22S–S26. 10.14219/jada.archive.2006.0406 17035672

[B49] Valdivia-Tapia A. C. Botelho J. N. Tabchoury C. P. M. Ricomini-Filho A. P. Giacaman R. A. Cury J. A. (2021). Fluoride bioavailability on demineralized enamel by commercial mouth rinses. Braz Dent. J. 32, 45–54. 10.1590/0103-6440202104475 34787250

[B50] Vieira-Junior W. F. Ferraz L. N. Giorgi M. C. C. Ambrosano G. M. B. Aguiar F. H. B. Lima D. A. N. L. (2019). Effect of mouth rinse treatments on bleached enamel properties, surface morphology, and tooth color. Oper. Dent. 44, 178–187. 10.2341/17-250-L 29953341

[B51] Weijden F. V. der. Sluijs E. V. der. Ciancio S. G. Slot D. E. (2015). Can chemical mouthrinse agents achieve plaque/gingivitis control? Dent. Clin. North Am. 59, 799–829. 10.1016/j.cden.2015.06.002 26427569

[B52] Winska K. Maczka W. Łyczko J. Grabarczyk M. Czubaszek A. Szumny A. (2019). Essential oils as antimicrobial agents - myth or real alternative? Molecules 24, 2130. 10.3390/molecules24112130 31195752 PMC6612361

[B53] Wiwattanarattanabut K. Choonharuangdej S. Srithavaj T. (2017). *In vitro* anti-cariogenic plaque effects of essential oils extracted from culinary herbs. J. Clin. Diagn Res. 11, DC30–DC35. 10.7860/JCDR/2017/28327.10668 29207708 PMC5713730

[B54] Yadav H. K. Yadav R. K. Chandra A. R. Thakkar R. (2016). The effectiveness of eucalyptus oil, orange oil, and xylene in dissolving different endodontic sealers. J. Conserv. Dent. 19, 332–337. 10.4103/0972-0707.186447 27563181 PMC4979279

[B55] Zero D. Zhang J. Harper D. Wu M. Kelly S. Waskow J. (2004). The remineralizing effect of an essential oil fluoride mouthrinse in an intraoral caries test. J. Am. Dent. Assoc. 135, 231–237. 10.14219/jada.archive.2004.0157 15005441

[B56] Zhang X. Z. Anderson P. Dowker S. E. P. Elliott J. C. (2000). Optical profilometric study of changes in surface roughness of enamel during *in vitro* demineralization. Caries Res. 34, 164–174. 10.1159/000016585 10773635

